# Editorial: Social cognition and mental health among children and youth

**DOI:** 10.3389/fpsyg.2023.1228526

**Published:** 2023-06-20

**Authors:** Kuiyun Zhi, Ling-Xiang Xia, David Bueno, Yongjin Chen, Zuoshan Li, Carlos Laranjeira

**Affiliations:** ^1^School of Public Policy and Administration, Chongqing University, Chongqing, China; ^2^China Public Service Evaluation and Research Center, Chongqing University, Chongqing, China; ^3^Faculty of Psychology, Southwest University, Chongqing, China; ^4^Key Laboratory of Cognition and Personality of Ministry of Education, Southwest University, Chongqing, China; ^5^Biomedical, Evolutionary, and Developmental Genetics Section, Faculty of Biology, University of Barcelona, Barcelona, Spain; ^6^Key Laboratory of Applied Psychology, Chongqing Normal University, Chongqing, China; ^7^School of Education, Chongqing Normal University, Chongqing, China; ^8^School of Health Sciences, Polytechnic of Leiria, Leiria, Portugal; ^9^Centre for Innovative Care and Health Technology (ciTechCare), Polytechnic of Leiria, Leiria, Portugal; ^10^Comprehensive Health Research Centre, University of Évora, Évora, Portugal

**Keywords:** children, youth, gender, class-related differences, social cognition, mental health, COVID-19 pandemic

This editorial comment on “*Social cognition and mental health among children and youth*” aims to provide a forum to improve research in this field and its contribution to health psychology, the understanding of risk and protective factors, and the exploration of innovative psychosocial interventions to benefit children and youth's health and wellbeing. People's feelings and social experiences are very influenced by their social, cultural, educational and autobiographical contexts.

Long-term effects of stress, such as COVID-19, have the potential to seriously endanger developing children's and youth's social cognition and mental health (Marques de Miranda et al., 2020; Theberath et al., 2022). Given the sanitary precautions requiring social isolation, school closures, and online lessons at home during the COVID-19 pandemic, which lasted for over 3 years, the risk of anxiety and depression disorders among children and adolescents rose (Senft et al., 2022). Young people's social cognition (such as beliefs, desires, self-concept, emotions, and academic achievement) and negative mental health outcomes (such as social isolation, sleep deprivation, smartphone addiction, impulse buying, and aggressive behavior) were likely significantly impacted by these restrictions.

Studies on the negative impact of the pandemic on young people have concentrated on mental health symptoms like anxiety, depression, distress, and subpar academic performance, among others (Elharake et al., 2023). Scientific societies have looked for effective post-pandemic recovery, preventive and intervention measures. Focusing on young people's physical health, psychological wellbeing, resilience, and social capital, some researchers contend that the COVID-19 crisis may also provide a chance for intergenerational family solidarity and personal growth (Moss et al., 2023). The pandemic has therefore need for deeper knowledge of social cognition and mental health among children and young people.

The present 18 academic papers focused on children, adolescents, and emerging adults, mainly from China, and included one study from Netherlands. Published in different article formats—namely Hypothesis and Theory, and empirical papers of varying research designs (such as cross-sectional, longitudinal, and intervention studies), the 18 articles on this Research Topic highlight the individual and contextual factors that can affect the mental health and psychological wellbeing of these age-groups. In this section, we refer briefly to the themes and novel contributions of these 18 articles.

Zeng et al. characterized post-traumatic growth and academic burnout and “the moderating role of core belief challenge among adolescents in an ethnic minority area in China during the COVID-19 pandemic” (p. 1). Fang et al. explored an audience's emotional experience and sharing of audio-visual artistic works during the COVID-19 pandemic. In a sample of college students, Chen S. et al. “explored the mediation and moderation effects on the relationship between different social media usage patterns, emotional responses, and consumer impulse buying during the COVID-19 pandemic” (p. 1). Lin et al. surveyed a large sample of Chinese adolescents and, based on health-related correlates, revealed the important role of negative problem orientation. Feng and Zhang explored “the effect of perceived teacher support and peer relationships on the mental health of Chinese university students, examining the mediating effects of reality and Internet altruistic behaviors on these relationships” (p. 1). Yue et al. explored the influence of peer actual appraisals on moral self-representations through peers' reflected appraisals among Chinese adolescents aged 12–14. Li M. et al. examined the relationship between empathy and altruistic behavior and their underlying mechanisms in Chinese undergraduate and graduate students. Yan et al. offered a theoretical model of how perceived control and sense of power affect adolescents' acceptance intention of intelligent online services through their perceived usefulness. Zhu et al. conducted behavioral and event-related potential experiments in China to illustrate “how young females with facial dissatisfaction process different levels of facial attractiveness” (p. 1). Chen X. et al. “revealed the effect of family cohesion on adolescents' engagement in school bullying and its mechanism of action, providing a theoretical basis for preventing and reducing the occurrence of school bullying incidents” (p. 1). Li A. et al. examined how a father's presence affects an adolescent's social responsibility, and their quality of interpersonal relationships. Li T. et al. “constructed a moderated chain mediation model to investigate the influence of childhood psychological abuse on relational aggression among Chinese adolescents” (p. 1). Zhang et al. demonstrated that photographic intervention could effectively improve positive affect and mitigate negative affect of college students during the COVID-19 pandemic. Wang et al. “showed that subjective wellbeing was positively correlated with social trust, trust in people, self-compassion, and social empathy” (p. 1), in a sample of first-generation Chinese college students. Hua and Zhou examined the relationship between personality assessment and mental health, via sequential mediation path involving the Barnum effect and ego identity. Liu et al. used a multi-methods approach “to analyze the mediating effects of social comparison and body image on social media selfie behavior and social anxiety in Chinese youth group” (p. 1). Fan et al. explored how relationship-maintenance strategies affect burnout in adolescent athletes, “including the potential mediating effects of the coach–athlete relationship and basic psychological needs satisfaction” (p. 1). Lastly, van Grieken et al. evaluated the longitudinal association between life events occurring before the second year and the risk of psychosocial problems at 3 years of age in Netherlands.

The papers on this Research Topic show how understanding the fundamental cognitive/social processes and the applied/clinical situations may help unify and expand our knowledge of Social Cognition and Mental Health throughout childhood and adolescence. However, the research herein is not exhaustive, and results must be weighed against the many conceptual and methodological constraints indicated in the publications. There are also geographic and cultural limits since most of the investigations were done in China. However, we believe that by offering an overview of the topic and emphasizing the most recent achievements, this Research Topic/eBook will be useful to both beginners and specialists toward improving the mental health of disadvantaged children and youth around the globe.

## Author contributions

All authors listed have made a substantial, direct, and intellectual contribution to the work and approved it for publication.

## References

[B1] Elharake J. A. Akbar F. Malik A. A. Gilliam W. Omer S. B. (2023). Mental health impact of COVID-19 among children and college students: a systematic review. Child Psychiatry Hum. Dev. 54, 913–925. 10.1007/s10578-021-01297-1 35013847 PMC8747859

[B2] Marques de Miranda D. da Silva Athanasio B. Sena Oliveira A. C. Simoes-E-Silva A. C. (2020). How is COVID-19 pandemic impacting mental health of children and adolescents? Int. J. Disaster Risk Reduct. 51, 101845. 10.1016/j.ijdrr.2020.101845 32929399 PMC7481176

[B3] Moss S. J. Mizen S. J. Stelfox M. Mather R. B. FitzGerald E. A. Tutelman P.. (2023). Interventions to improve well-being among children and youth aged 6-17 years during the COVID-19 pandemic: a systematic review. BMC Med. 21, 131. 10.1186/s12916-023-02828-4 37013542 PMC10069351

[B4] Senft B. Liebhauser A. Tremschnig I. Ferijanz E. Wladika W. (2022). Effects of the COVID-19 pandemic on children and adolescents from the perspective of teachers. Front. Educ. 7, 808015. 10.3389/feduc.2022.808015

[B5] Theberath M. Bauer D. Chen W. Salinas M. Mohabbat A. B. Yang J.. (2022). Effects of COVID-19 pandemic on mental health of children and adolescents: a systematic review of survey studies. SAGE Open Med. 10, 20503121221086712. 10.1177/20503121221086712 35371484 PMC8972920

